# Supplementary material for: A Schematic Approach to Defining the Prevalence of COL VI Variants in Five Years of Next-Generation Sequencing
Source: Int J Mol Sci. 2022 Nov 23;23(23):14567. doi: 10.3390/ijms232314567 (PMC9735635; doi:10.3390/ijms232314567)

Supplementary Method and Figures

Immunofluorescent labeling of fibroblasts

After 3 days of incubation with L-ascorbic acid phosphate (50 ug/mL), cells were fixed in 4% paraformaldehyde for 10 minutes at room temperature in the absence (**S1**) or presence (**S2**) of 0.1% Triton X-100 (Sigma 21123). Triton X-100 permeabilizes the cells and is used to assay intracellular retention of collagen VI. The cells were then subjected to blocking reagent for 1 hour (20% fetal bovine serum in 1X PBS) and then primary antibodies (1:100 dilution of rabbit polyclonal anti– collagen VI [Abcam ab6588] diluted in blocking reagent) was applied to the cells at 4°C overnight. Secondary antibodies (Alexafluor 488 goat anti-rabbit [Invitrogen A11034], diluted 1:1000 in blocking reagent) and the nuclear marker DAPI (4′,6-diamidino-2-phenylindole, diluted 1:1000 in blocking reagent), were applied for 1 hour at room temperature. Slides were mounted and cells were visualized with a Nikon Ti2-E inverted microscope a 20x objective and an FITC/DAPI filter set. Labeled cultures were compared with the normal control to pinpoint rarefication in the amount of collagen VI and intracellular retention within fibroblast cells (magnifying inserts in panel S2). Scale bars 100 μm

S1 not permeabilized fibroblasts (4% PFA)

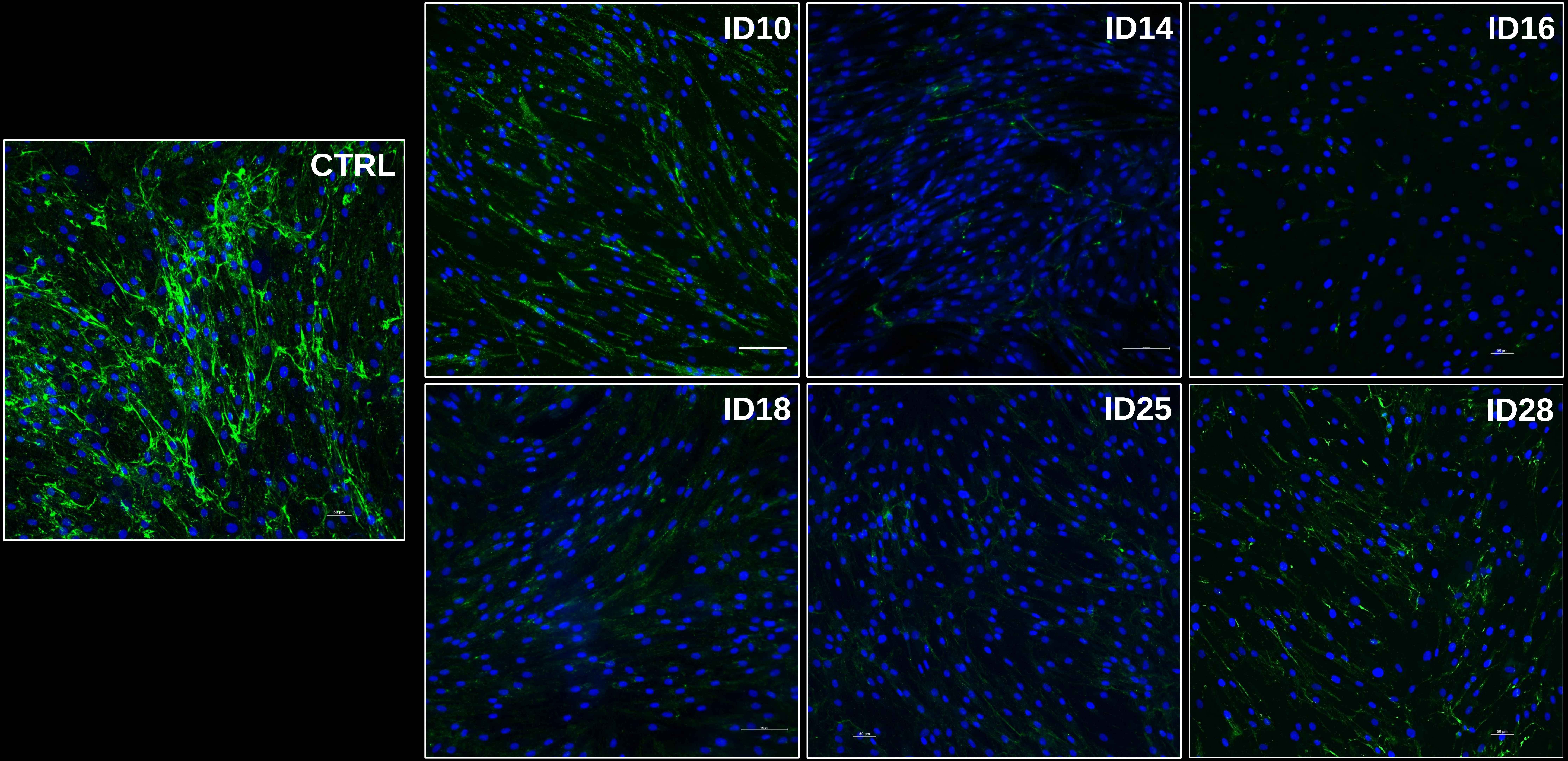

Collagen VI / DAPI

S2 permeabilized fibroblasts (4% PFA + 0,1% TritonX-100)

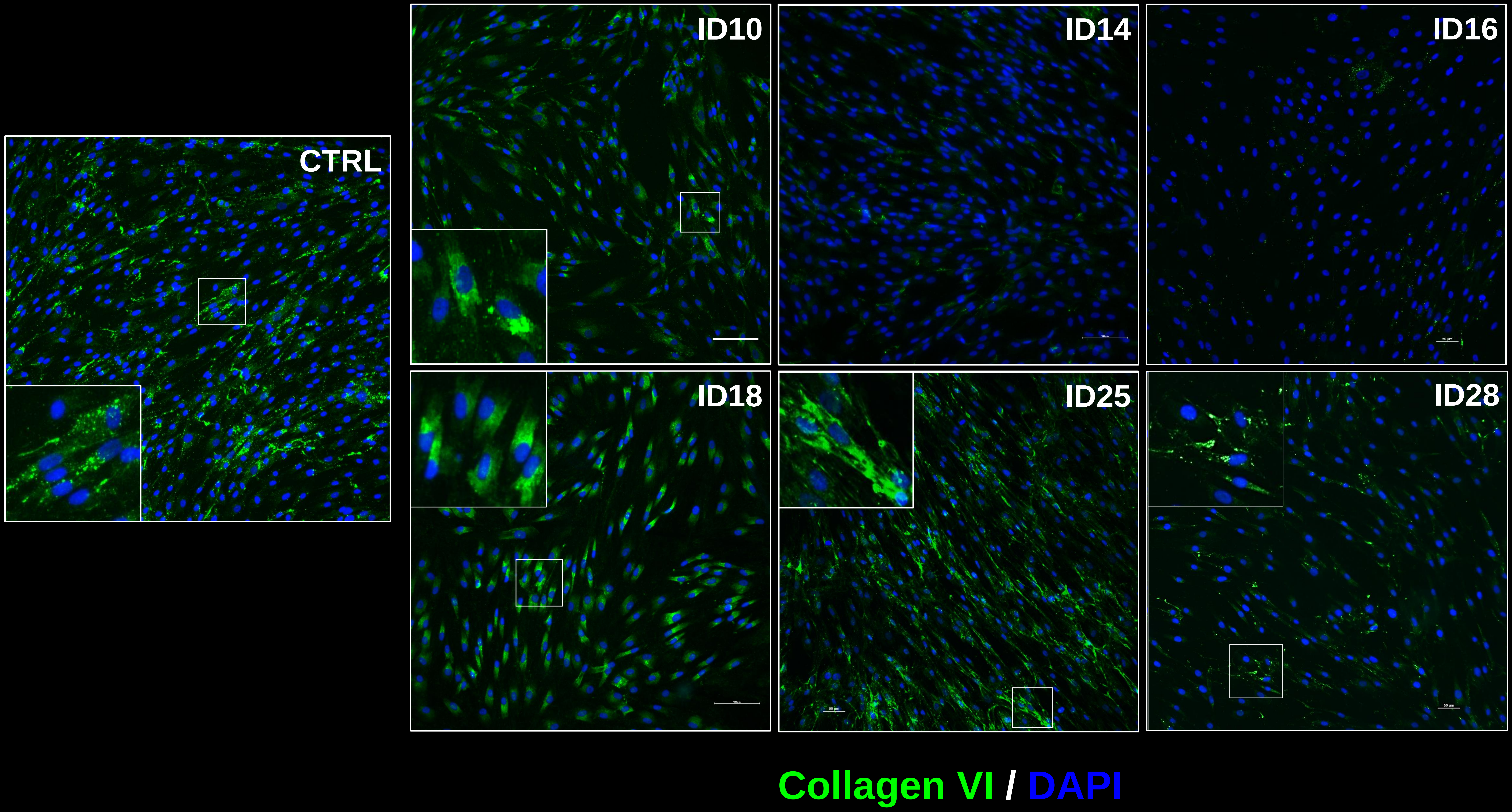

Supplement: Supplementary file 1 [file ijms-23-14567-s001.zip › ijms-1974619-supplementary.pdf]
